# Harnessing lentiviral vectors for in vivo gene therapy of liver metastases

**DOI:** 10.1002/ctm2.1542

**Published:** 2024-01-17

**Authors:** Giovanna Giacca, Luigi Naldini, Mario Leonardo Squadrito

**Affiliations:** ^1^ Targeted Cancer Gene Therapy Unit San Raffaele Telethon Institute for Gene Therapy IRCCS San Raffaele Scientific Institute Milan Italy; ^2^ Vita Salute San Raffaele University Milan Italy

**Keywords:** cancer immunotherapy, gene therapy, lentiviral vectors, liver metastasis

1

Liver metastases (LMS) commonly arise from gastrointestinal tumors, such as colorectal cancer (CRC) and pancreatic cancer (PDAC). The immunosuppressive environment of the liver favors cancer cell seeding and metastasis generation by impairing immune activation and promoting immune escape. In agreement with this observation, most pharmacological interventions, including recent immunotherapies, prove ineffective in the presence of LMS. For example, immunotherapeutic treatments, which are successful against melanoma, show reduced efficacy in the presence of LMS.^1^ Thus, it is of pivotal importance to develop innovative therapeutic interventions that favor immune activation and unleash immune effector functions to eradicate LMS.

To respond to this unmet clinical need, we developed a lentiviral vector (LV)‐based in vivo gene therapy platform, which upon a single well‐tolerated intravenous (i.v.) injection in mice, conveys expression of interferon‐alpha (IFNα) to liver macrophages.^2^ LVs are HIV‐1‐derived self‐inactivating viral vectors, which do not replicate in the host upon cell transduction. Moreover, LVs efficiently transduce both proliferating as well as non‐proliferating cells and integrate into the genome of the target cell enabling long‐term IFNα output. IFNα is an immune‐activating, pro‐apoptotic and antiangiogenic cytokine, which can be employed therapeutically on LMS. By employing distinct mouse models of CRC and PDAC LMS, we showed that sustained expression of IFNα from liver macrophages reduced the growth of established LMS up to complete eradication in some mice. Response to treatment was associated with increased antigen presentation by macrophages and dendritic cells, as well as augmented infiltration and activation of CD8^+^ T cells (Figure 1). Moreover, IFNα from engineered macrophages protected mice against the seeding and growth of metastatic cancer cells infused in the circulation, indicating that the platform besides reducing the growth of established LMS confers protection against the formation of new metastatic foci.

**FIGURE 1:**
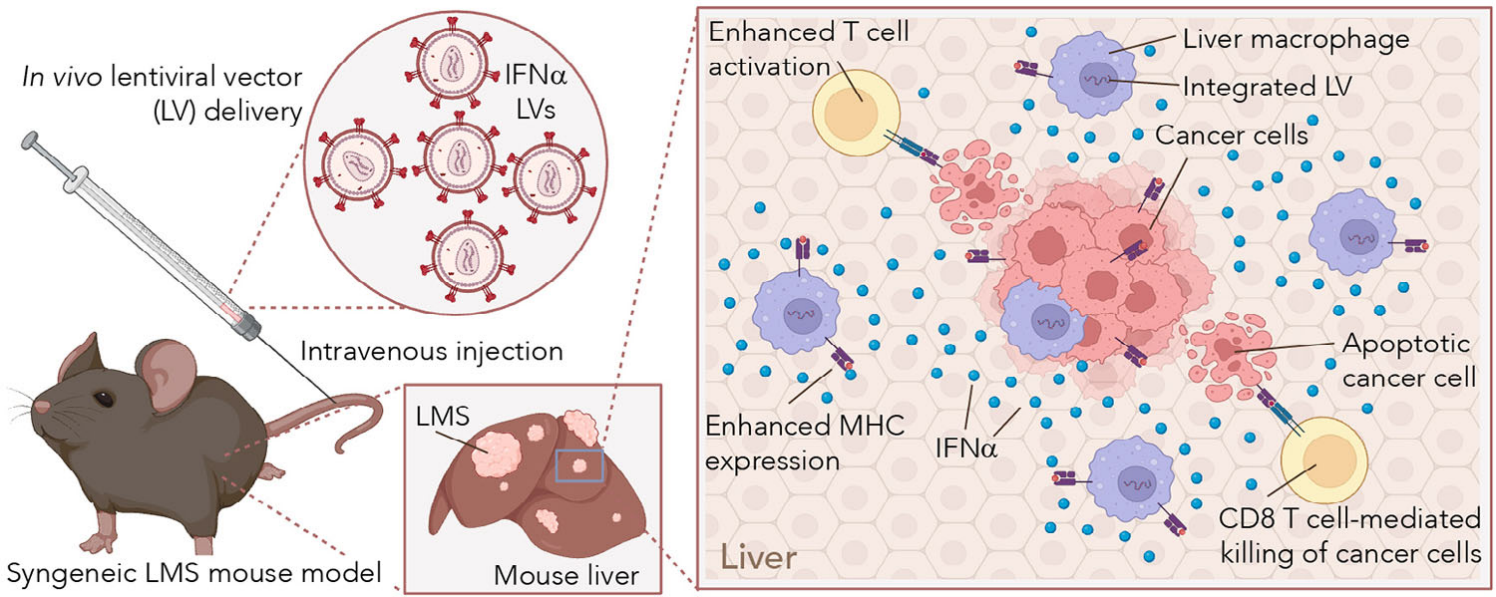
Schematics showing lentiviral vector (LV)‐based gene therapy of liver metastases (LMS) in mice. An LV driving the expression of interferon‐alpha (IFNα) is injected intravenously in the tail vein of an immunocompetent mouse‐bearing established LMS. LVs efficiently engineer liver macrophages, which in turn release IFNα in proximity to LMS, leading to enhanced antigen presentation and activation of CD8^+^ T cell effector activity.

Interestingly, resistance to the treatment in some mice was associated with (i) increased interleukin‐10 signaling, which is associated with immune suppressive functions; (ii) increased expression of the immune inhibitory receptor cytotoxic T‐lymphocyte associated protein 4 (CTLA‐4); and (iii) infiltration of immunosuppressive type I regulatory CD4^+^ T cells. A combination of gene‐based IFNα delivery with monoclonal antibodies blocking CTLA‐4 resulted in a strong therapeutic response, leading to LMS eradication in most mice. Consistent with this resistance mechanism, we detected elevated levels of CTLA‐4 expression in LMS from the fraction of CRC patients followed in our institution who exhibited high endogenous IFNα signaling. This result underscores the role of CTLA‐4 in negatively regulating IFNα‐induced immune activation.

LVs can be pseudotyped with envelope glycoproteins different from the one originally found in HIV‐1. In our work, we utilized LVs pseudotyped with vesicular stomatitis virus glycoprotein (VSV‐G). When administered systemically to mice and non‐human primates, VSV‐G‐pseudotyped LVs transduce almost exclusively the liver and spleen.^3^ Within the liver the most transduced cell types are macrophages, hepatocytes and liver sinusoidal endothelial cells (LSECs), in order of abundance; within the spleen, which is transduced to a lesser extent, red pulp macrophages are the most abundant transduced cell type. The biodistribution of VSV‐G LVs upon i.v. administration is most likely a result of the physiological filtering activity of the two organs and the high phagocytic activity and histological localization of phagocytic cells within these organs. Therefore, to drive selective transgene expression to macrophages in proximity to liver metastases, we incorporated in the LV design transcriptional and post‐transcriptional regulatory features such as (i) a macrophage‐specific promoter and (ii) microRNA (miRNA) target sites. The promoter employed was selected from the *Mrc1* gene, which is upregulated upon M2‐like signals, such as those present in tumors and LMS. Furthermore, the LV design incorporated sequences with perfect complementarity to miRNAs miR‐122‐5p and miR‐126‐3p, which prevent transgene expression in off‐target hepatic populations expressing the corresponding miRNA, namely hepatocytes and LSECs.^4^, ^5^ This LV configuration (i.e. the choice of the promoter and miRNA target sequences) favored preferential transgene expression, and thus IFNα signaling, in liver areas in proximity to cancer cells.

Recombinant IFNα has been thoroughly tested for its antitumoral effect in several clinical trials over various tumor types, showing mixed therapeutic benefits, and being abandoned in some applications due to its side effects.^6^ Recombinant IFNα received clinical approval for the treatment of chronic hepatitis B and C, hairy cell leukemia, myelogenous chronic leukemia and Kaposi's sarcoma, among others.^7^, ^8^, ^9^, ^10^ Of note, IFNα recombinant protein is characterized by a short half‐life, leading to peak‐and‐trough pharmacokinetics, which is associated with desensitization and toxicity. Conversely, in murine models, local release by genetically engineered macrophages was characterized by continuous and stable plasma concentrations, resulting virtually in the absence of toxicity. In fact, we did not observe histopathological abnormalities, increased transaminase levels or development of autoreactive antibodies upon treatment of mice. Translation to patients could also benefit from the availability of mitigation strategies to be employed in case of side effects, such as anti‐IFNα monoclonal antibodies. Moreover, it may be possible to further engineer the LV to incorporate inducible cassettes within the LV design or the expression of a constitutive suicide gene, enabling it to switch off the treatment at any desired time.^11^

This study highlights the therapeutic benefits of LVs harnessed in vivo to treat LMS. Compared to other viral platforms, preexisting immunity against LV particles is limited to HIV‐infected people, supporting the feasibility of harnessing LVs systemically in patients. Of note, despite the clinical applications of LVs to engineer hematopoietic stem cells (HSCs) and T cells *ex vivo*, in vivo clinical applications of LVs are still missing. In recent years, improvements in vector design as well as choice of regulatory sequences, such as endogenous promoters, have contributed to addressing initial concerns regarding genotoxicity and thus advance LV application as well as safety (Figure 2).^12^ Up to date, hundreds of patients with rare genetic diseases, including pediatric patients, have been successfully treated with LV‐based treatments obtaining unforeseen clinical benefits.^13^ LV‐based therapies, such as Libmeldy, Skysona, Zynteglo and Lyfgenia, used to treat distinct genetic disorders, have recently received European Medicines Agency or Food and Drug Administration approval. (https://www.ema.europa.eu/en/medicines/human/EPAR/libmeldy, https://www.fda.gov/vaccines‐blood‐biologics/skysona, https://www.fda.gov/vaccines‐blood‐biologics/zynteglo, https://www.fda.gov/vaccines‐blood‐biologics/lyfgenia). Moreover, clinically available immunotherapy treatments based on the use of chimeric antigen receptor (CAR)‐T cells, such as Kymriah and Breyanzi, leverage LVs to achieve stable transgene CAR expression on T cells.^14^, ^15^ Besides CAR‐T cell applications, LVs are being tested in first‐in‐human trials to convey IFNα to glioblastoma multiforme upon ex vivo engineering of HSCs (ClinicalTrials.gov ID NCT03866109).^16^ All these recent applications of LVs support the translational potential of LVs harnessed in vivo paving the way for their clinical application on patients with LMS.

**FIGURE 2:**
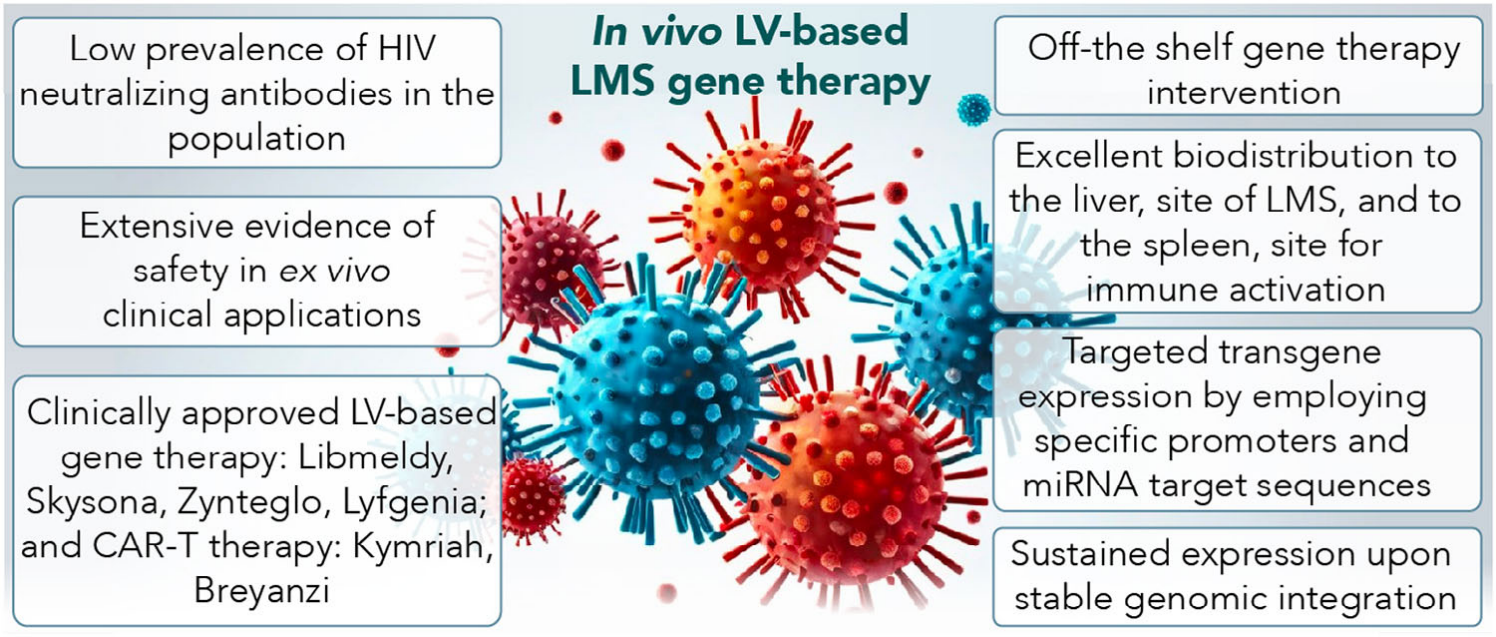
Schematics highlighting key features of lentiviral vector (LV)‐based gene therapy of liver metastases (LMS).

## AUTHOR CONTRIBUTIONS

G.G., L.N. and M.L.S. conceptualized and wrote the commentary.

## CONFLICT OF INTEREST STATEMENT

Luigi Naldini and Mario Leonardo Squadrito are inventors of a patent on KC‐directed gene transfer and Luigi Naldini is an inventor of patents on miRNA‐regulated LV technology filed and managed by the San Raffaele Scientific Institute and the Telethon Foundation.

## ETHICS STATEMENT

Not Applicable.
